# Spanish Pre-Olympic Athletes’ Motivations and Barriers to Pursuing Dual Career as a Function of Sociodemographic, Sport and Academic Variables

**DOI:** 10.3389/fpsyg.2022.850614

**Published:** 2022-04-25

**Authors:** Adrián Mateo-Orcajada, Alejandro Leiva-Arcas, Raquel Vaquero-Cristóbal, Lucía Abenza-Cano, Juan Alfonso García-Roca, Lourdes Meroño, Emanuele Isidori, Antonio Sánchez-Pato

**Affiliations:** ^1^Olympic Studies Centre, Murcia, Spain; ^2^Faculty of Sport, Catholic University San Antonio of Murcia, Murcia, Spain; ^3^Department of Movement, Human and Health Sciences, Università degli Studi di Roma Foro Italico, Rome, Italy

**Keywords:** academic performance, student-athletes, scholarships, sport modality, sport performance

## Abstract

The dual career allows elite athletes to attain their maximum competitive and academic performance, but the COVID-19 pandemic hindered their development and changed their perception of the importance given to the sporting and educational environment. For this reason, the aim of the present study was to determine the differences in the motivations and perceived barriers, the importance given to academic qualifications, and the perception of the dual career from a multifactorial perspective, of elite athletes according to sex, type of sport practiced, job performance, time of sports career, type of athlete, and type of scholarship received. A total of 100 student-athletes participated in the research study by completing the “Perceptions of dual career student-athletes” questionnaire. The results showed that athletes from individual modalities (*p* = 0.012) and those who did not receive any scholarships described more barriers (*p* < 0.001). In addition, women studied more because they enjoyed it (*p* = 0.007); athletes from individual modalities studied to work later (*p* = 0.008); athletes who do not work perceived a greater influence between study and sports performance (*p* = 0.029); at the beginning and at the best stage of their sports career, a greater influence of academics on performance was perceived (*p* = 0.016); and athletes who considered themselves professionals, and athletes who did not receive any scholarships (*p* = 0.025), reported that the conciliation between sports and academic life was difficult (*p* = 0.034). The results obtained point to the importance of dual career scholarships for student-athletes, as well as the need for the programs implemented for these athletes to consider sex, sport modality or type of scholarship granted.

## Introduction

Elite athletes invest a great part of their sporting life training to achieve success in their sports, which entails great economic and time demands that limit their development in other areas outside of sports (Aquilina, 2013). Despite the sacrifices made, the economic reward is small, and most of these athletes cannot subsist solely on the income generated by their sports career (Aquilina, 2013; Martínez-Abajo et al., 2020). Therefore, alternatives such as the dual academic-sports career become more important, as athletes can simultaneously and complementarily flourish in sports and academics, which favors their holistic development and better adaptation to post-sports life (Stambulova and Wylleman, 2015; Abelkalns et al., 2021).

The benefits of the dual career have been analyzed in previous studies, highlighting the easiness found by student-athletes in their process of insertion into the working world, being more satisfactory than that of athletes who focus exclusively on their sport, and increasing their chances of attaining a stable future after finishing their sports career (Torregrosa et al., 2015; Jordana et al., 2017; Barriopedro et al., 2018; Harrison et al., 2020). In the psychological domain, student-athletes also report benefits, since engaging in another activity that allows them to escape from the stressful environment of competition helps them build a multidimensional identity that facilitates their retirement from elite sports, as opposed to the unidimensional, exclusively sporting identity that seems to hinder the transition to retirement (Guirola Gómez et al., 2018; Gavala-González et al., 2019; Defruyt et al., 2020; Moreno et al., 2020; Felix-Mena et al., 2021). An increase in the intrinsic motivation needed to continue studying and competing when they receive institutional scholarships has also been found (Guirola Gómez et al., 2018; Gavala-González et al., 2019), and student-athletes seem to have a higher perception of social support than non-students (Fuchs et al., 2016; Harrison et al., 2020). Beyond the psychological benefits, the dual career offers athletes numerous possibilities for personal, financial, social and health development, allowing them to acquire different skills that are transferable to different areas of their lives (Graczyk et al., 2017). Therefore, the dual career is understood as an occupation that provides educational and sporting benefits and results in the comprehensive and balanced development of the athlete, which facilitates their adaptation to different situations of daily life, as well as the process of sporting retirement (Guirola Gómez et al., 2018; De Subijana et al., 2021).

These reasons make the dual career a highly valued alternative among elite athletes due to the sporting, educational and personal benefits it brings, but there are still numerous barriers that hinder its development (De Subijana et al., 2015; Gavala-González et al., 2019). Among the main barriers, athletes point out the lack of flexibility to adapt academic and sports schedules (Fuchs et al., 2016; da Costa et al., 2020; De Subijana et al., 2021); the difficulty in managing their time (De Subijana et al., 2015); the impossibility of attending classes due to long-term sports gatherings (Guirola Gómez et al., 2018; Gavala-González et al., 2019); and the existing physical distance between the university and the training center (Guirola Gómez et al., 2018).

Although benefits and barriers are the most evaluated elements by athletes to start a dual career, research conducted in recent years has shown that factors such as gender, sports level, the scenarios they face, the competencies they have, sports modality or perceived support, can also be relevant in the enrollment and development of the dual career (Sánchez Pato et al., 2018; Perez-Rivases et al., 2020; Zou et al., 2020), although few studies have specifically focused on these aspects. Regarding gender, women have lower expectations than men regarding their sport performance, so they attach more importance to the dual career, and place more value on the possibility of working in a job related to their academic degree (Fuchs et al., 2016; Tekavc and Erpic, 2018; De Subijana et al., 2021). Considering the sports level, professional athletes experience greater difficulties in reconciling sports and academic life, and perceive their integration into the workplace more negatively than amateur athletes (De Subijana et al., 2018). Regarding the sports modality, there is controversy in this area, and it is unknown whether it is individual or team athletes who have more difficulties (Tekavc et al., 2015; Fuchs et al., 2016; Graczyk et al., 2017; Condello et al., 2019; De Subijana et al., 2020). Finally, with respect to perceived aid, the athletes who obtain a scholarship to study complete their studies to a greater extent than those who do not have any aid (Coelho et al., 2021).

Therefore, it is not possible to generalize the difficulties and benefits of achieving dual career success without first conducting a specific analysis of the context in which the dual career takes place (Kuettel et al., 2017). In this sense, it is especially important to evaluate the environment in which the dual career is carried out, since previous research has shown that public and private sports centers and education centers offer different possibilities of development to dual career athletes, which is a determining factor for the athletic and academic development of the student-athlete (Mejías et al., 2021). This is even more true after the global pandemic caused by COVID-19, as previous studies have shown that the student-athletes’ perception of the importance and barriers to dual career success changed after the pandemic, affecting their academic and athletic preferences (Abenza-Cano et al., 2020; Izzicupo et al., 2021; Woodford and Bussey, 2021). This situation produced changes in the lifestyle of these student-athletes, who had to establish new personal goals, adapt their daily routines and grant more importance to their dual career (Woodford and Bussey, 2021), which was reflected in increased study hours and a lower intention to continue with their sports career once they finished their studies, as compared to athletes who completed their dual career before the pandemic (Abenza-Cano et al., 2020).

However, no studies have analyzed the motivations and perceived barriers of university student-athletes associated to a dual career once competitive normalcy was restored after the COVID-19 pandemic, compounded by the lack of previous research on contextual factors that might affect student-athletes’ dual career perceptions. Previous research conducted on junior athletes has shown the importance of analyzing the needs, barriers, challenges, and resources available to dual career athletes for the development of coping skills and general aspects of these athletes’ lives (López-Flores et al., 2021). Therefore, research is needed to determine which aspects are most important for dual-career university athletes in the aftermath of the COVID-19 pandemic, so that professionals working in this field understand the real needs of student-athletes and develop individualized programs to meet their demands. For this reason, the main objective of the present study was to determine the differences in the motivations and perceived barriers, the importance given to academic qualifications, and the perception of the dual career of elite university athletes, from a multifactorial perspective according to sex, type of sport practiced, sport self-classification, stage of sports career, type of athlete, and type of scholarship received.

## Materials and Methods

### Design

The study design was descriptive and cross-sectional, and a non-probability convenience sampling method was used. The STROBE statement (Vandenbroucke et al., 2014) was followed for the study design and development of the manuscript. Study participants provided their consent to participate prior to data collection and were informed of the study objectives and the confidentiality of the data obtained during the study. The institutional ethics committee reviewed and authorized the protocol designed for data collection, in accordance with the guidelines from the World Medical Association (code:19/6/2015).

### Participants

The sample size was calculated using Rstudio 3.15.0 software (Rstudio Inc., United States). The significance level was set at α = 0.05. The standard deviation (SD) was *SD* = 0.9 considering previous studies (De Subijana et al., 2021). With an estimated error (d) of 0.23, the required sample size for a 99% confidence interval (CI) was 100 subjects.

The inclusion criteria were (a) being in the database of pre-Olympic athletes of the Spanish Olympic Committee for the Tokyo 2020 games; (b) being considered a high-level athlete according to the Spanish definition of High for Sports and being included in the list published in the Official State Bulletin (BOE); (c) having resumed normal training and competition after the COVID-19 pandemic and (d) being currently enrolled in a university degree or master’s degree within a dual-career university program.

The size of the sample universe was 231 individuals. The final sample consisted of 100 (43.29% participation rate) Spanish pre-Olympic student-athletes (41% men and 59% women) with an average age of 24.86 ± 5.99 years. Of these, 4% of the athletes were enrolled in their 1st year, 21% in their 2nd year, 21% in their third, 13% in their 4th year, 17% in their 5th year, 12% in their 6th year, and 12% had been at university for more than 6 years. All the participants were part of the Spanish pre-Olympic team for Tokyo 2020 (Olympic Games held in 2021) and participated in the research on a completely voluntary basis, without receiving any type of compensation. When the sample was divided according to the type of sport practiced, 61% practiced individual modalities and 39% practiced team modalities. According to their sports self-classification, 53% considered themselves professionals, 32% semi-professionals and 15% amateurs. Regarding the stage of their sporting career, 30% were in their initial stage, 40% in their peak performance stage, and 30% at the end of their sporting career. In addition to competing and studying, 40% were working and 60% were not. Regarding the scholarships they received, 48% did not receive any type of scholarship from the university, 28% received a partial tuition scholarship and 24% received a full tuition scholarship.

### Procedure

The “Perceptions of dual career student-athletes” (ESTPORT) questionnaire was utilized, a validated questionnaire used in previous research (Sánchez-Pato et al., 2016; Gavala-González et al., 2019; Abenza-Cano et al., 2020). It allows measuring the perception of student-athletes regarding their dual career. The complete questionnaire consists of 84 items, and was completed in its entirety by the participants. The questionnaire has a high internal consistency, as the Cronbach’s alpha coefficients were higher than 0.70 for the complete questionnaire, with an alpha value of 0.81 for the “academic career” construct, 0.73 for the “sports career” construct and 0.83 for the “barriers” dimension, which is within acceptable limits (Corbetta, 2007; Sánchez-Pato et al., 2016; Conde et al., 2021).

Socio-demographic and contextual items were analyzed to obtain information about tsex (item 1) with a dichotomous answer; employment status (item 14) with a dichotomous answer; type of sport (item 5) with a short answer, which was divided into individual and team sports according to the definitions of Sebastiani (1994) for individual sports and Parlebas (2001) for team sports; stage of sport career (item 8), with multiple-choice answers; sport self-classification (item 7), with multiple-choice answers; and type of scholarship obtained (item 11), with multiple-choice answers.

To measure the barrier dimension, following the methodology by Conde et al. (2021), items 26 to 37 used a Likert scale response option, ranging from 1 (“strongly disagree”) to 5 points (“strongly agree”). To obtain the score for the “perceived barriers” dimension, the mean of the scores obtained in items 26 to 37 of the questionnaire was calculated following the methodology by Conde et al. (2021). The dimensions of “aid tools” and “sports mentoring” established by Conde et al. (2021) were not taken into consideration following the methodology of De Subijana et al. (2021), because the athletes belonged to different universities and sports centers, so the responses to these items were greatly heterogeneous, which could condition the perception of the dual careers of these athletes (Mejías et al., 2021).

Regarding motivations, the questionnaire refers to reasons to study (item 15), and to expectations upon graduation (item 23), in its dual career construct (Sánchez-Pato et al., 2016), with multiple-choice questions. The questionnaire also presents a series of items regarding academic and athletic performance (items 16 to 18, and 20), and the rate of academic year completion (item 40). In these questions there were dichotomous response items (items 16 to 18); Likert scale items with five options, from 1: very easy to 5: very difficult (item 20); and multiple response items (item 40).

As for the “importance given to qualifications” dimension, only question 48 was taken into consideration, following the methodology of De Subijana et al. (2021), who modified and validated the ESTPORT questionnaire by exclusively including this item to assess the importance that athletes gave of grades in the dual career. This question used Likert scale response options from 1 (“strongly disagree”) to 5 (“strongly agree”).

The student-athletes were contacted via email to participate in the study through the Spanish Olympic Committee. First, the participants completed and signed the informed consent form, where they were informed about the objectives and procedure of the study, and subsequently, they completed the questionnaire anonymously and individually, without academic or competitive pressure, and without the presence of their coaches or teachers. The participants did not receive any extra indications or explanations about the purpose of the questionnaire, other than that indicated in the questionnaire itself. The questionnaire was disseminated through the GoogleForms^®^ platform and the participants completed it in 20-30 min.

### Statistical Analysis

The normality of the data was initially assessed with the Kolmogorov-Smirnov test, homogeneity with the Levene’s test, and sphericity with the Mauchly test. All the variables included in the analysis showed a normal distribution, so parametric tests were performed. The descriptive analysis of quantitative variables showed mean values and standard deviations, while frequencies and percentages were calculated for qualitative variables. The Student’ *t*-test for independent samples was performed to find the existing differences in the scores of “perceived barriers” and “importance given to qualifications” as a function of sex, type of sport practiced, and the athlete’s job. Cohen’s d was calculated to establish the effect size (ES) in these cases, defined as small when *d* < 0.2; moderate when *d* < 0.8; and large when *d* > 0.8 (Cohen, 1988). For the analysis of the differences in the perceived barriers and the importance given to the qualifications as a function of the stage of the sports career, the type of athlete and the scholarship received, a one-way analysis of variance (ANOVA) was used, carrying out the Bonferroni pairwise comparison in the variables with statistical significance, adjusting for the value of *p* < 0.016. Partial eta squared (η^2^) was used to calculate the effect size (ES), and was defined as small: ES ≥ 0.10; moderate: ES ≥ 0.30; large: ES ≥ 1.2; very large: ES ≥ 2.0 (Hopkins et al., 2009). The chi-square analysis (χ^2^) made it possible the establishment of the differences in the questions related to the reasons why athletes study, academic and sports performance, and expectations after completing their studies, according to sex, type of sport practiced, work performed, stage of sports career, sport self-classification and type of scholarship received. Cramer’s V was used for the *post hoc* comparison of the 2 × 2 tables, and the contingency coefficient was used in the 2 × n tables, to obtain the statistical value. The maximum expected value was 0.707; *r* < 0.3 indicated a low association; *r* < 0.5 indicated a moderate association; and *r* > 0.5 indicated a high association (Cramér, 1946). The *p* < 0.05 value was set to determine statistical significance. The statistical analysis was performed using the SPSS statistical package (v.25.0; SPSS Inc., IL, United States).

## Results

Tables 1, 2 show the perceived barriers and the importance of the grades obtained by the student-athletes according to sex, type of sport practiced, work performed, stage in the sports career, sport self-classification and type of scholarship received. The results showed significant differences in the perceived barriers, according to the type of sport practiced, with the athletes in individual modalities perceiving more barriers (individual: 2.67 ± 0.76; team: 2.26 ± 0.80; *p* = 0.012), with a moderate effect size; and to the type of scholarship received (*p* < 0.001), with the athletes without any type of scholarship showing more barriers (none: 2.82 ± 0.64; partial: 2.36 ± 0.90; full: 2.06 ± 0.72; *p* < 0.001), with a small effect size. There were also differences in the importance given to grades when considering the type of scholarship received (*p* = 0.036), with athletes with full tuition discount giving more importance to grades (none: 2.97 ± 0.68; partial: 3.33 ± 0.90; full: 3.39 ± 0.63; *p* = 0.036), with a small effect size (Table 1).

**TABLE 1:** Overall score on the items perceived barriers and importance given to the grades (from 1 to 5) by student athletes according to sex, employment status, type of sport, stage of sport career, sport self-classification, and type of scholarship obtained.

SEX	Males (*n* = 41)	Females (*n* = 59)		*t*; *p*	*d*
Perceived barriers (Mean ± SD)	2.59 ± 0.75	2.44 ± 0.83		0.942; *p* = 0.348	0.19
Importance of grades (Mean ± SD)	3.11 ± 0.72	3.21 ± 0.78		−0.645; *p* = 0.520	0.13

**EMPLOYMENT STATUS**	**Working (*n* = 40)**	**Not working (*n* = 60)**		***t*; *p***	**d**

Perceived barriers (Mean ± SD)	2.59 ± 0.85	2.45 ± 0.76		0.819; *p* = 0.415	0.17
Importance of grades (Mean ± SD)	3.18 ± 0.91	3.17 ± 0.64		0.057; *p* = 0.954	0.01

**TYPE OF SPORT**	**Individual (*n* = 61)**	**Team (*n* = 39)**		***t*; *p***	**d**

Perceived barriers (Mean ± SD)	2.67 ± 0.76	2.26 ± 0.80		2.546; *p* = 0.012*	0.52
Importance of grades (Mean ± SD)	3.08 ± 0.77	3.31 ± 0.71		−1.472; *p* = 0.144	0.31

**STAGE OF SPORT CAREER**	**Start (*n* = 30)**	**Best moment (*n* = 40)**	**End (*n* = 30)**	***F*; *p***	**Effect size**

Perceived barriers (Mean ± SD)	2.44 ± 0.71	2.50 ± 0.83	2.60 ± 0.86	0.299; *p* = 0.742	0.006
Importance of grades (Mean ± SD)	3.06 ± 0.66	3.23 ± 0.80	3.20 ± 0.80	0.462; *p* = 0.632	0.010

**SPORT SELF-CLASIFICATION**	**Professional (*n* = 53)**	**Semi-professional (*n* = 31)**	**Amateur (*n* = 16)**	***F*; *p***	**Effect size**

Perceived barriers (Mean ± SD)	2.41 ± 0.83	2.65 ± 0.72	2.59 ± 0.83	0.956; *p* = 0.388	0.020
Importance of grades (Mean ± SD)	3.11 ± 0.74	3.33 ± 0.76	3.07 ± 0.80	1.019; *p* = 0.365	0.021

**TYPE OF SCHOLARSHIP**	**None (*n* = 48)**	**Partial (*n* = 28)**	**Full (*n* = 23)**	***F*; *p***	**Effect size**

Perceived barriers (Mean ± SD)	2.82 ± 0.64	2.36 ± 0.90	2.06 ± 0.72	8.972; *p* < 0.001**	0.157
Importance of grades (Mean ± SD)	2.97 ± 0.68	3.33 ± 0.90	3.39 ± 0.63	3.433; *p* = 0.036*	0.067

*p < 0.05; **p < 0.001.

**TABLE 2:** Perceived barriers and importance given to grades according to type of sport and type of scholarship obtained.

Barrier	Type of sport			*t*; *p*	*d*
	Individual (*n* = 61)	Team (*n* = 39)			
I find myself unable to balance study and training time	2.31 ± 1.03	1.62 ± 0.82		1.493; *p* = 0.001	0.75

**Barrier**	**Type of scholarship**			***F*; *p***	**Effect size**
	**None (*n* = 49)**	**Partial (*n* = 28)**	**Full (*n* = 23)**		

The university is far from my training site	2.96 ± 1.53	2.00 ± 1.56	2.09 ± 1.31	4.776; *p* = 0.011*	0.090
The cost of education is high	3.37 ± 1.42	3.07 ± 1.74	1.57 ± 0.90	12.910; *p* < 0.001**	0.210
I do not have enough university support	3.37 ± 1.22	2.71 ± 1.53	1.61 ± 1.03	14.858; *p* < 0.001**	0.235
Student schedules are not flexible	3.24 ± 1.54	2.96 ± 1.48	1.61 ± 0.94	10.912; *p* < 0.001**	0.184

**Importance given to grades**	**Type of sport**			***t*; *p***	***d***
	**Individual (*n* = 61)**	**Team (*n* = 39)**			

I get more satisfaction from getting high grades in a subject than winning a game in my sport	2.43 ± 1.20	2,13 ± 1,12		1.216; *p* = 0.227	0,26

**Importance given to grades**	**Type of scholarship**			***F*; *p***	**Effect size**
	**None (*n* = 49)**	**Partial (*n* = 28)**	**Full (*n* = 23)**		

I get more satisfaction from getting high grades in a subject than winning a game in my sport	2.34 ± 1.15	2.44 ± 1.40	2.09 ± 0.99	0.592; *p* = 0.555	0.012

*p < 0.05; **p < 0.001.

The specific barriers that showed statistically significant differences according to sex, employment status, type of sport, time of sports career, sport self-classification and type of scholarship obtained, are shown in Table 2. It should be noted that only the barrier “I find myself unable to balance study and training time” showed significant differences between individual and team sports (*p* = 0.001), with individual sports men and women having the most difficulties (individual sport: 2.31 ± 1.03; team sport: 1.62 ± 0.82). When considering the type of scholarship received, the barriers were higher in athletes who did not receive any help in “the university is far from my training site” (*p* < 0.011), “the cost of education is high” (*p* < 0.001), “I do not have enough university support” (*p* < 0.001) and “student schedules are not flexible” (*p* < 0.001), with a small effect size in all cases. No differences were found for any individual barrier according to sex, employment status, time in the sports career, or sport self-classification. Regarding the importance given to the grades obtained, the differences were not significant in “I obtain more satisfaction from getting a high grade in a subject than from winning a game in my sport” (*p* = 0.555).

Differences were found in the reasons why the student-athletes studied, questions related to academic and sports performance, and expectations at the end of their studies, according to sex (Table 3), type of sport practiced (Table 3), employment situation (Table 4), stage in their sports career (Table 4), sport self-classification (Table 5), and type of scholarship received (Table 5).

**TABLE 3:** Motives for study, academic and athletic performance, and expectations upon completion of studies as a function of sex and type of sport.

		Sex				Type of sport			
		Male	Female	Group differences (χ^2^, p)	Cramer’s V/contingency coefficient	Individual	Collective	Group differences (χ^2^, p)	Cramer’s V/contingency coefficient
**Reason for studying**									
To increase my job prospects	Yes	35(85.4%)	49(83.1%)	χ^2^ = 0.096; *p* = 0.756	0.031	56(91.8%)	28(71.8%)	χ^2^ = 7.086; *p* = 0.008*	0.266
	No	6(14.6%)	10(16.9%)			5(8.2%)	11(28.2%)		
Because I enjoy studying and want to educate myself	Yes	13(31.7%)	35(59.3%)	χ^2^ = 7.390; *p* = 0.007*	0.272	27(44.3%)	21(53.8%)	χ^2^ = 0.875; *p* = 0.349	0.094
	No	28(68.3%)	24(40.7%)			34(55.7%)	18(46.2%)		
For social interaction	Yes	3(7.3%)	4(6.8%)	χ^2^ = 0.011; *p* = 0.917	0.010	5(8.2%)	2(5.1%)	χ^2^ = 0.344; *p* = 0.557	0.059
	No	38(92.7%)	55(93.2%)			56(91.8%)	37(94.9%)		
I have always wanted to study	Yes	2(4.9%)	5(8.5%)	χ^2^ = 0.481; *p* = 0.488	0.069	5(8.2%)	2(5.1%)	χ^2^ = 0.344; *p* = 0.557	0.059
	No	39(95.1%)	54(91.5%)			56(91.8%)	37(94.9%)		
For financial assistance	Yes	0(0.0%)	1(1.7%)	χ^2^ = 0.702; *p* = 0.402	0.084	0(0.0%)	1(2.6%)	χ^2^ = 1.580; *p* = 0.209	0.126
	No	41(100.0%)	58(98.3%)			61(100.0%)	38(97.4%)		
**Academic and athletic performance**									
Studies interfere with athletic performance	Yes	31(75.6%)	31(52.5%)	χ^2^ = 5.463; *p* = 0.019*	0.234	39(63.9%)	23(59.0%)	χ^2^ = 0.248; *p* = 0.618	0.050
	No	10(24.4%)	28(47.5%)			22(36.1%)	16(41.0%)		
Athletic performance interferes with studies	Yes	32(78.0%)	40(67.8%)	χ^2^ = 1.261; *p* = 0.261	0.112	44(72.1%)	28(71.8%)	χ^2^ = 0.001; *p* = 0.971	0.004
	No	9(22.0%)	19(32.2%)			17(27.9%)	11(28.2%)		
Do you consider yourself to be…	Student-Athlete	18(43.9%)	23(39.0%)	χ^2^ = 0.242; *p* = 0.623	0.049	27(44.3%)	14(35.9%)	χ^2^ = 0.688; *p* = 0.407	0.083
	Athlete-Student	23(56.1%)	36(61.0%)			34(55.7%)	25(64.1%)		
Difficulty in balancing sports and academic life	Very easy	3(7.3%)	0(0.0%)	χ^2^ = 8.540; *p* = 0.074	0.280	1(1.6%)	2(5.1%)	χ^2^ = 8.079; *p* = 0.089	0.273
	Easy	3(7.3%)	7(11.9%)			3(4.9%)	7(17.9%)		
	Neither easy nor difficult	10(24.4%)	21(35.6%)			18(29.5%)	13(33.3%)		
	Difficult	22(53.7%)	22(37.3%)			29(47.5%)	15(38.5%)		
	Very difficult	3(7.3%)	9(15.3%)			10(16.4%)	2(5.1%)		
**Academic and athletic performance**									
Rate of academic year completion	1 year/level	24(58.5%)	33(55.9%)	χ^2^ = 0.797; *p* = 0.850	0.089	32(52.5%)	25(64.1%)	χ^2^ = 8.079; *p* = 0.089	0.273
	2 year/level	16(39.0%)	23(39.0%)			25(41.0%)	14(35.9%)		
	3 year/level	1(2.4%)	2(3.4%)			3(4.9%)	0(0.0%)		
	4 year/level	0(0.0%)	1(1.7%)			1(1.6%)	0(0.0%)		
**Expectations upon graduation**									
Continue studying	Yes	13(31.7%)	24(40.7%)	χ^2^ = 0.835; *p* = 0.361	0.091	23(37.7%)	14(35.9%)	χ^2^ = 0.033; *p* = 0.855	0.018
	No	28(68.3%)	35(59.3%)			38(62.3%)	25(64.1%)		
Work	Yes	29(70.7%)	44(74.6%)	χ^2^ = 0.181; *p* = 0.670	0.043	47(77.0%)	26(66.7%)	χ^2^ = 1.301; *p* = 0.254	0.114
	No	12(29.3%)	15(25.4%)			14(23.0%)	13(33.3%)		
Continue sports career	Yes	25(61.0%)	31(52.5%)	χ^2^ = 0.698; *p* = 0.403	0.084	30(49.2%)	26(66.7%)	χ^2^ = 2.952; *p* = 0.086	0.172
	No	16(39.0%)	28(47.5%)			31(50.8%)	13(33.3%)		
Do not know	Yes	3(7.3%)	3(5.1%)	χ^2^ = 0.214; *p* = 0.644	0.046	2(3.3%)	4(10.3%)	χ^2^ = 2.054; *p* = 0.152	0.143
	No	38(92.7%)	56(94.9%)			59(96.7%)	35(89.7%)		

*p < 0.05.

**TABLE 4:** Motives for study, academic and athletic performance, and expectations at the end of studies as a function of employment status and the stage of athletic career.

		Employment status				Stage of athletic career				
		Working	Not working	Group differences (χ^2^, p)	Cramer’s V/contingency coefficient	Start	Best stage	End	Group differences (χ^2^, p)	Cramer’s V/contingency coefficient
**Reason for studying**										
To increase my job prospects	Yes	35(87.5%)	49(81.7%)	χ^2^ = 0.608; *p* = 0.436	0.078	24(77.4%)	35(87.5%)	25(86.2%)	χ^2^ = 1.469; *p* = 0.480	0.120
	No	5(12.5%)	11(18.3%)			7(22.6%)	5(12.5%)	4(13.8%)		
Because I enjoy studying and want to educate myself	Yes	17(42.5%)	31(51.7%)	χ^2^ = 0.808; *p* = 0.369	0.090	15(48.4%)	21(52.5%)	12(41.4%)	χ^2^ = 0.836; *p* = 0.658	0.091
	No	23(57.5%)	29(48.3%)			16(51.6%)	19(47.5%)	17(58.6%)		
For social interaction	Yes	4(10.0%)	3(5.0%)	χ^2^ = 0.922; *p* = 0.337	0.096	1(3.2%)	2(5.0%)	4(13.8%)	χ^2^ = 2.980; *p* = 0.225	0.170
	No	36(90.0%)	57(95.0%)			30(96.8%)	38(95.0%)	25(86.2%)		
I have always wanted to study	Yes	4(10.0%)	3(5.0%)	χ^2^ = 0.922; *p* = 0.337	0.096	3(9.7%)	3(7.5%)	1(3.4%)	χ^2^ = 0.919; *p* = 0.632	0.095
	No	36(90.0%)	57(95.0%)			28(90.3%)	37(92.5%)	28(96.6%)		
For financial assistance	Yes	0(0.0%)	1(1.7%)	χ^2^ = 0.673; *p* = 0.412	0.082	0(0.0%)	1(2.5%)	0(0.0%)	χ^2^ = 1.515; *p* = 0.469	0.122
	No	40(100.0%)	59(98.3%)			31(100.0%)	39(97.5%)	29(100.0%)		
**Academic and athletic performance**										
Studies interfere with athletic performance	Yes	23(57.5%)	39(65.0%)	χ^2^ = 0.573; *p* = 0.449	0.076	25(80.6%)	24(60.0%)	13(44.8%)	χ^2^ = 8.272; *p* = 0.016*	0.276
	No	17(42.5%)	21(35.0%)			6(19.4%)	16(40.0%)	16(55.2%)		
Athletic performance interferes with studies	Yes	24(60.0%)	48(80.0%)	χ^2^ = 4.762; *p* = 0.029*	0.218	25(80.6%)	30(75.0%)	17(58.6%)	χ^2^ = 3.903; *p* = 0.142	0.194
	No	16(40.0%)	12(20.0%)			6(19.4%)	10(25.0%)	12(41.4%)		
Do you consider yourself to be…	Student-Athlete	18(45.0%)	23(38.3%)	χ^2^ = 0.441; *p* = 0.507	0.066	20(64.5%)	9(22.5%)	12(41.4%)	χ^2^ = 12.748; *p* = 0.002*	0.336
	Athlete-Student	22(55.0%)	37(61.7%)			11(35.5%)	31(77.5%)	17(58.6%)		
Difficulty in balancing sports and academic life	Very easy	1(2.5%)	2(3.3%)	χ^2^ = 3.039; *p* = 0.551	0.172	1(3.2%)	2(5.0%)	0(0.0%)	χ^2^ = 6.386; *p* = 0.604	0.245
	Easy	4(10.0%)	6(10.0%)			4(12.9%)	4(10.0%)	2(6.9%)		
	Neither easy nor difficult	16(40.0%)	15(25.0%)			6(19.4%)	12(30.0%)	13(44.8%)		
	Difficult	14(35.0%)	30(50.0%)			15(48.4%)	17(42.5%)	12(41.4%)		
	Very difficult	5(12.5%)	7(11.7%)			5(16.1%)	5(12.5%)	2(6.9%)		
**Academic and athletic performance**										
Rate of course completion	1 year/level	22(55.0%)	35(58.3%)	χ^2^ = 1.619; *p* = 0.655	0.126	23(74.2%)	25(62.5%)	9(31.0%)	χ^2^ = 14.540; *p* = 0.024*	0.356
	2 year/level	16(40.0%)	23(38.3%)			8(25.8%)	14(35.0%)	17(58.6%)		
	3 year/level	1(2.5%)	2(3.3%)			0(0.0%)	1(2.5%)	2(6.9%)		
	4 year/level	1(2.5%)	0(0.0%)			0(0.0%)	0(0.0%)	1(3.4%)		
**Expectations upon graduation**										
Continue studying	Yes	18(45.0%)	19(31.7%)	χ^2^ = 1.830; *p* = 0.176	0.135	12(38.7%)	15(37.5%)	10(34.5%)	χ^2^ = 0.122; *p* = 0.941	0.035
	No	22(55.0%)	41(68.3%)			19(61.3%)	25(62.5%)	19(65.5%)		
Work	Yes	32(80.0%)	41(68.3%)	χ^2^ = 1.657; *p* = 0.198	0.129	21(67.7%)	28(70.0%)	24(82.8%)	χ^2^ = 2.019; *p* = 0.364	0.141
	No	8(20.0%)	19(31.7%)			10(32.3%)	12(30.0%)	5(17.2%)		
Continue sports career	Yes	20(50.0%)	36(60.0%)	χ^2^ = 0.974; *p* = 0.324	0.099	22(71.0%)	23(57.5%)	11(37.9%)	χ^2^ = 6.698; *p* = 0.035*	0.251
	No	20(50.0%)	24(40.0%)			9(29.0%)	17(42.5%)	18(62.1%)		
Do not know	Yes	2(5.0%)	4(6.7%)	χ^2^ = 0.118; *p* = 0.731	0.034	2(6.5%)	3(7.5%)	1(3.4%)	χ^2^ = 0.506; *p* = 0.777	0.071
	No	38(95.0%)	56(93.3%)			29(93.5%)	37(92.5%)	28(96.6%)		

*p < 0.05.

**TABLE 5:** Motives for study, academic and athletic performance, and expectations at the end of studies according to the sport self-classification and the type of scholarship.

		Sport self-classification					Type of scholarship				
		Professional	Semi-professional	Amateur	Group differences (χ^2^, p)	Cramer’s V/contingency coefficient	None	Partial	Full	Group differences (χ^2^, p)	Cramer’s V/contingency coefficient
**Reason for studying**											
To increase my job prospects	Yes	44(83.0%)	26(83.9%)	14(87.5%)	χ^2^ = 0.184; *p* = 0.912	0.043	41(83.7%)	22(78.6%)	21(91.3%)	χ^2^ = 1.531; *p* = 0.465	0.123
	No	9(17.0%)	5(16.1%)	2(12.5%)			8(16.3%)	6(21.4%)	2(8.7%)		
Because I enjoy studying and want to educate myself	Yes	29(54.7%)	11(35.5%)	8(50.0%)	χ^2^ = 2.929; *p* = 0.231	0.169	15(30.6%)	20(71.4%)	13(56.5%)	χ^2^ = 12.762; *p* = 0.002*	0.336
	No	24(45.3%)	20(64.5%)	8(50.0%)			34(69.4%)	8(28.6%)	10(43.5%)		
For social interaction	Yes	4(7.5%)	1(3.2%)	2(12.5%)	χ^2^ = 1.446; *p* = 0.485	0.119	4(8.2%)	1(3.6%)	2(8.7%)	χ^2^ = 0.709; *p* = 0.702	0.084
	No	49(92.5%)	30(96.8%)	14(87.5%)			45(91.8%)	27(96.4%)	21(91.3%)		
I have always wanted to study	Yes	2(3.8%)	4(12.9%)	1(6.3%)	χ^2^ = 2.521; *p* = 0.284	0.157	7(14.3%)	0(0.0%)	0(0.0%)	χ^2^ = 7.834; *p* = 0.020*	0.270
	No	51(96.2%)	27(87.1%)	15(93.8%)			42(85.7%)	28(100.0%)	23(100.0%)		
For financial assistance	Yes	1(1.9%)	0(0.0%)	0(0.0%)	χ^2^ = 0.896; *p* = 0.639	0.094	0(0.0%)	0(0.0%)	1(4.3%)	χ^2^ = 3.382; *p* = 0.184	0.181
	No	52(98.1%)	31(100.0%)	16(100.0%)			49(100.0%)	28(100.0%)	22(95.7%)		
**Academic and athletic performance**											
Studies interfere with athletic performance	Yes	26(49.1%)	26(83.9%)	10(62.5%)	χ^2^ = 10.064; *p* = 0.007*	0.302	36(73.5%)	19(67.9%)	7(30.4%)	χ^2^ = 12.870; *p* = 0.002*	0.338
	No	27(50.9%)	5(16.1%)	6(37.5%)			13(26.5%)	9(32.1%)	16(69.6%)		
Athletic performance interferes with studies	Yes	40(75.5%)	22(71.0%)	10(62.5%)	χ^2^ = 1.050; *p* = 0.592	0.102	41(83.7%)	17(60.7%)	14(60.9%)	χ^2^ = 6.494; *p* = 0.039*	0.247
	No	13(24.5%)	9(29.0%)	6(37.5%)			8(16.3%)	11(39.3%)	9(39.1%)		
Do you consider yourself to be…	Student-Athlete	12(22.6%)	18(58.1%)	11(68.8%)	χ^2^ = 16.210; *p* < 0.001**	0.373	22(44.9%)	10(35.7%)	9(39.1%)	χ^2^ = 0.664; *p* = 0.717	0.081
	Athlete-Student	41(77.4%)	13(41.9%)	5(31.2%)			27(55.1%)	18(64.3%)	14(60.9%)		
**Academic and athletic performance**											
Difficulty in balancing sports and academic life	Very easy	0(0.0%)	2(6.5%)	1(6.3%)	χ^2^ = 16.628; *p* = 0.034*	0.378	1(2.0%)	2(7.1%)	0(0.0%)	χ^2^ = 17.526; *p* = 0.025*	0.386
	Easy	4(7.5%)	6(19.4%)	0(0.0%)			1(2.0%)	5(17.9%)	4(17.4%)		
	Neither easy nor difficult	18(34.0%)	7(22.6%)	6(37.5%)			13(26.5%)	6(21.4%)	12(52.2%)		
	Difficult	26(49.1%)	9(29.0%)	9(56.3%)			26(53.1%)	12(42.9%)	6(26.1%)		
	Very difficult	5(9.4%)	7(22.6%)	0(0.0%)			8(16.3%)	3(10.7%)	1(4.3%)		
Rate of course completion	1 year/level	23(43.4%)	22(71.0%)	12(75.0%)	χ^2^ = 16.810; *p* = 0.010*	0.379	28(57.1%)	18(64.3%)	11(47.8%)	χ^2^ = 4.766; *p* = 0.574	0.213
	2 year/level	28(52.8%)	9(29.0%)	2(12.5%)			20(40.8%)	9(32.1%)	10(43.5%)		
	3 year/level	2(3.8%)	0(0.0%)	1(6.3%)			1(2.0%)	1(3.6%)	1(4.3%)		
	4 year/level	0(0.0%)	0(0.0%)	1(6.3%)			0(0.0%)	0(0.0%)	1(4.3%)		
**Expectations upon graduation**											
Continue studying	Yes	22(41.5%)	8(25.8%)	7(43.8%)	χ^2^ = 2.441; *p* = 0.295	0.154	19(38.8%)	8(28.6%)	10(43.5%)	χ^2^ = 1.334; *p* = 0.513	0.115
	No	31(58.5%)	23(74.2%)	9(56.3%)			30(61.2%)	21(71.4%)	13(56.5%)		
Work	Yes	36(67.9%)	29(93.5%)	8(50.0%)	χ^2^ = 11.628; *p* = 0.003*	0.323	36(73.5%)	18(64.3%)	19(82.6%)	χ^2^ = 2.162; *p* = 0.339	0.145
	No	17(32.1%)	2(6.5%)	8(50.0%)			13(26.5%)	10(35.7%)	4(17.4%)		
Continue sports career	Yes	23(43.4%)	21(67.7%)	12(75.0%)	χ^2^ = 7.496; *p* = 0.024*	0.264	31(63.3%)	19(67.9%)	6(26.1%)	χ^2^ = 11.000; *p* = 0.004*	0.315
	No	30(56.6%)	10(32.3%)	4(25.0%)			18(36.7%)	9(32.1%)	17(73.9%)		
Do not know	Yes	4(7.5%)	0(0.0%)	2(12.5%)	χ^2^ = 3.402; *p* = 0.182	0.181	2(4.1%)	2(7.1%)	2(8.7%)	χ^2^ = 0.681; *p* = 0.711	0.082
	No	49(92.5%)	31(100.0%)	14(87.5%)			47(95.9%)	26(92.9%)	21(91.3%)		

*p < 0.05; **p < 0.001.

Regarding sex, women studied more to a greater extent than men, because they enjoyed it and wanted to improve their education (*p* = 0.007), and thought that their studies did not interfere as much with their sports performance as compared to men (*p* = 0.019) (Table 3). The value of the contingency coefficient was low in both cases (Table 3).

When comparing athletes in individual and team sports, it was found that a significantly higher percentage of athletes in individual sports studied to increase their chances of finding work, as compared to those in collective modalities (*p* = 0.008), with a low contingency coefficient (Table 3).

It should also be noted that the student-athletes who did not work perceived a greater interference between sports performance and studies, as compared to the student-athletes who worked (*p* = 0.0029). In this case, the value of the contingency coefficient was low (Table 4).

Regarding the athlete-students’ stage of their sports career, those who were at the beginning or at the best stage of their sports career perceived a greater interference of studies with performance (*p* = 0.016), with a low contingency coefficient. It is also relevant that the student-athletes who were at the beginning of their sports career perceived themselves as student-athletes, while those who were in their best stage or in their final stage perceived themselves as athlete-students in a higher percentage (*p* = 0.002), with a moderate value for the contingency coefficient. Regarding the rate of passing academic year per calendar year, it was found that while the majority of the student-athletes who were at the beginning or at the best stage of their sports career passed one academic year per calendar year, the student-athletes who were at the end of their sports career passed one academic year every 2 years (*p* = 0.024), with the value of the contingency coefficient being moderate in both cases. With respect to the expectations that the student-athletes had at the end of their studies, it was found that a higher percentage of the student-athletes who were at the beginning and in the best stage of their career had the intention of continuing their sports career, as compared to those who were at the end of their career (*p* = 0.035), with a low contingency coefficient (Table 4).

Table 5 shows the differences between the student-athletes who considered themselves to be professionals, semi-professionals and amateurs, with the results being especially relevant in the dimension of academic and sports performance. Statistically, a higher percentage of semi-professional student-athletes considered that studying interfered with sports performance, followed by amateur athletes, while professional athletes did not have the same perspective (*p* = 0.007), with the value of the contingency coefficient being moderate. However, half of the student-athletes who considered themselves professionals and amateurs stated that the conciliation between sports and academic life was difficult (*p* = 0.034), with a moderate contingency coefficient. It should also be noted that the majority of the student-athletes who considered themselves professionals needed 2 years to pass each academic year, while semi-professionals and amateurs did so in one (*p* = 0.010), with a moderate contingency coefficient value. In addition, professional athletes considered themselves as athlete-students, as compared to semi-professionals and amateurs who considered themselves as student-athletes (*p* < 0.001) to a greater extent, with a moderate contingency coefficient. Regarding expectations after finishing their studies, student-athletes who considered themselves professionals and semi-professionals had a greater intention to look for a job (*p* = 0.003), with the value of the contingency coefficient being moderate, while amateur athletes had a greater intention to continue with their sports career (*p* = 0.024), with a low contingency coefficient.

The student-athletes also showed significant differences in their perception of the dual career according to the type of scholarship received. Most of the student-athletes who received a partial enrollment scholarship studied because they enjoyed it and wanted to improve their level of education (*p* = 0.002), with a moderate contingency coefficient value. The percentage of student-athletes who indicated “I have always wanted to study” as a reason for studying was significantly lower among those on partial or full scholarships (*p* = 0.020), with a low contingency coefficient. Student-athletes who did not receive any type of scholarship perceived a greater influence of their studies on performance (*p* = 0.002), with a moderate contingency coefficient value, and of performance on studies (*p* = 0.039), with a low contingency coefficient value, as well as greater difficulty in reconciling sports and academic life (*p* = 0.025), as compared with scholarship athletes, with a moderate value for the contingency coefficient. However, these student-athletes, and those receiving a partial scholarship, had higher expectations of continuing their sports career after completing their studies, than those receiving full scholarships (*p* = 0.004), with the contingency coefficient being moderate in this case (Table 5).

## Discussion

The influence exerted by the COVID-19 pandemic on the motivations, barriers, and perceptions of university student-athletes of Olympic modalities was one of the main objects of study of the research study, which determined differences in the motivations and perceived barriers, the importance given to academic qualifications, and the perception of the dual career of elite athletes, from a multifactorial perspective according to sex, type of sport practiced, sport self-classification, stage of sports career, type of athlete, and type of scholarship received. Previous research conducted on dual-career students enrolled in high school and university studies during COVID-19 lockdown showed a significant decrease in the time devoted to studies and sport, but academic and sport commitments were determinant in coping with the pandemic and maintaining an active lifestyle (Izzicupo et al., 2021). Once the lockdown was overcome and with the new academic and sports normality, the results obtained in the present research showed that the most determinant barriers for university student-athletes during the dual career were related to the remoteness of the university to the training center, the lack of flexibility in academic schedules, or the lack of support from the university. This is similar to previous studies conducted in high school students (López-Flores et al., 2021), which could be explained by the lack of sports mentoring in both settings. In addition, during lockdown, dual career students pointed out the convenience provided by virtual teaching, which meant being able to combine academic and sports performance (Abenza-Cano et al., 2020; Izzicupo et al., 2021). Therefore, these findings should be taken into account in the design of future dual career programs if adequate academic and sports performance is to be achieved.

With respect to sex, the reasons why athletes decided to start a dual career showed significant differences, with women studying to a greater extent because they enjoyed it and wanted to improve their education, stating that their academic obligations did not interfere with their sporting performance. These results are similar to those found in previous research, where women had lower expectations regarding their sports career, placing greater importance on education to be able to obtain a job related to their academic degree (Fuchs et al., 2016; Tekavc and Erpic, 2018; De Subijana et al., 2021). The economic differences between sexes are still very present in the area of sports, which is reflected in a lower salary, financial support, and aid for female athletes (De Subijana et al., 2021), forcing them to seek alternatives such as an academic career, which is a very good option because it facilitates their insertion into the workplace. However, future studies would have to analyze more areas of the dual career in which differences may be found between sexes, to ensure that all athletes develop under the same sporting and academic conditions.

Considering sports modality, individual modality athletes showed more barriers than team modality athletes, which is similar to the results found in previous research, in which individual modality athletes received less support from academic staff (Fuchs et al., 2016). A possible explanation for these findings could be that individual modality athletes train more hours per week and stay at sport gatherings for longer periods of time than team modality athletes (De Subijana et al., 2020), which decreases their schedule flexibility and increases their perception of barriers in not being able to tend to the dual career demands. Another possible explanation could be that in team sports, the maximum performance and sport abandonment occur later, so the time available to develop the dual career is longer than in individual modalities, leaving them in a better position to face sports retirement and the transition to post-sport life (De Subijana et al., 2020). The importance of these results is greater when considering that elite athletes need an average of 2 years to successfully complete each academic year (De Subijana et al., 2021), with similar results to those found in the present study, so that athletes of individual modalities have a very reduced time frame to carry out the dual career, and it is therefore necessary to consider the sport modality in which the athlete participates for the design of future programs aimed at student-athletes.

In addition, the type of sport modality had a significant influence on the reasons why the athletes enrolled in the dual career, with the increased possibility of working being the most decisive reason for athletes in individual modalities. Previous research has shown that athletes in team modalities have a better economic and employment situation once their sporting career is over, perhaps due to the difference in sporting rewards and salaries compared to individual modalities, leaving them in a better position to face sporting retirement and the transition to post-sporting life (De Subijana et al., 2020). The athletes in individual sports must therefore look for a new occupation after retirement, since their sporting career has a shorter duration and provides less economic benefits than in team sports (Rosen and Sanderson, 2001). If these results were subsequently corroborated in other scientific studies, it would increase the value of the academic career and would become one of the main reasons for individual sportsmen and women to consider pursuing a dual career.

Surprisingly, it was observed that non-working student-athletes perceived a greater interference between sports performance and studying than working student-athletes. No previous studies have analyzed the perception of the dual career of athletes who work at the same time that they train and study, but these findings could be explained as a result of the substantial decrease in available time that student-athletes have when they start the dual career (Aquilina, 2013; Guirola Gómez et al., 2018). It is true that elite athletes who train and work experience an even more reduced time availability when they start studying, but they develop a greater number of competencies related to time management and adaptability that is transferred between work, academics, and sports (Crespo Celda and Crespo Dualde, 2016; Moyà et al., 2017; Moreno et al., 2021; Reyes-Hernández et al., 2021), as compared to athletes who only trained. Therefore, student-athletes who do not work suffer more drastic changes when starting the dual career because they must reorganize their available time in order to devote enough of it to achieve their academic goals. For this reason, it would be interesting for the dual career programs to carry out psychological interventions in which the athletes obtain the necessary coping resources to face the changes that will occur in their daily lives during their dual career.

Another relevant finding that had not been reported in previous research was that the student-athletes’ stage of their sports career was a determinant factor in their perception of the dual career. Student-athletes who were at the early and at the peak stages of their careers perceived a greater interference of their studies with their sports performance, although they were able to complete one academic year per year and perceived themselves as student-athletes to a greater extent, as compared to those in their final stage of their sports careers who passed one academic year every other year and perceived themselves as athlete-students. A possible explanation for these results could be that athletes in the initial stage of their sporting career are younger, are used to passing one academic year per calendar year and have a great uncertainty about their sporting future, with the level of demand being very high to maintain sporting and academic performance (Garcia Mas et al., 2003), and have an alternative option when their sports career ends; while older athletes who are in the final stage of their sports career try to enjoy their last years as elite athletes, perceiving studies as a complement, not as a priority, and have family obligations that hinder their dedication to both studies and sport (Moesch, 2013).

It should also be noted that the student-athletes who were at the beginning and at the best stage of their sports career had greater intentions to continue in the field of sports after finishing their studies, as compared to those who were in their final stages. This could be due to the fact that the demands to which athletes are subjected during their sports career, as well as injuries suffered, or poor relationships with teammates and coaches, are factors that demotivate older athletes (Reynaga-Estrada et al., 2017), and could lead to the abandonment of the sports career if these factors are strongly present during this stage. In addition, the level of education of the athlete’s close environment could be fundamental in this decision. Moreno et al. (2020) found that athletes who continued in the field of sports after completing their studies had parents who were not educated, while those who had parents who had received an education disengaged from the field of sports to a greater extent. Therefore, future research studies conducted with elite athletes who are in their final stage of their dual career should consider factors such as the close environment, injuries, emotional exhaustion, loss of motivation, or the relationship with coaches and teammates, as these factors could be determinants for the continuity of these athletes in the field of sports.

The perception of the dual career was also different depending on whether the student-athlete considered himself/herself professional, semi-professional or amateur, as semi-professional athletes perceived a greater influence of their studies on sports performance, but professionals perceived more difficulties in reconciling sports and academic life and needed 2 years to pass each academic year. Previous research showed similar results, with professional athletes having more difficulties in reconciling sport and academics because they were exclusively dedicated to their physical preparation, while amateurs equally distributed their time between them (De Subijana et al., 2018). These results can be explained by the fact that professional athletes have employment contracts that link them to the sports club or institution in which they practice the sport, which obliges them to meet a minimum sports performance based on the achievement of objectives during the season, for which they need greater dedication (Gómez et al., 2019). It would be interesting for future studies to analyze the differences in sports and academic performance between dual career athletes with a scholarship and those with a contract with a sports institution, since the preferences of these athletes are assumed to be different in either case and could influence performance in these areas.

The type of scholarship received by the athletes was also a factor to be considered in the perception of barriers and the importance given to academic grades, with athletes who did not receive any scholarships showing the most barriers. These results follow the line of previous research, in which athletes who obtained a scholarship to study completed their studies to a greater extent than those who did not receive any aid (Coelho et al., 2021). The financial reward received by the athletes during their athletic career is small (Aquilina, 2013), so paying for university tuition can be an added effort that few athletes can afford. Partial and full tuition scholarships provide athletes with considerable assistance in coping with their studies, but require a minimum annual academic performance to remain eligible (Milton et al., 2012; Pitts and Rezek, 2012). This could be the reason why athletes with scholarships perceived fewer barriers, as they do not have to pay the tuition with their sports income.

In addition, it was the athletes with scholarships who studied the most because they enjoyed it and wanted to improve their level of education, but it is surprising that the reason “I have always wanted to study” was the least important among the athletes, regardless of whether or not they received a scholarship. Even then, it was the non-scholarship athletes who indicated this motive in the highest percentage. These results coincide with those found in previous research, which showed that athletes with a scholarship were more involved in their studies and completed them to a greater extent than athletes without a scholarship (Coelho et al., 2021). It should be noted that elite athletes start practicing their sports modalities from very early ages, and attach more importance to sports performance than to academics, so that in their future intentions, their academic performance occupy a secondary place (Puig and Vilanova, 2006; Wylleman and Reints, 2010; Stambulova and Wylleman, 2019). However, as these athletes become older and perceive that their sports career, as their main source of income, is not enough to ensure a future after their retirement from sports, realize that they need to reorganize their priorities and increase their educational level to have a work alternative away from the field of sports (Puig and Vilanova, 2006; Aquilina, 2013; Knights et al., 2016).

It is also important to note that athletes who did not receive any type of scholarship had higher expectations of continuing their athletic career after completing their studies as compared to those who received a scholarship. Vickers and Morris (2021) indicated that student-athletes who finished university took different paths, one of which was to continue with their sports full time, which is consistent with the results obtained in the present research. It is possible that these results are due to the fact that athletes who had received previous aid showed higher levels of academic and sporting demands than athletes without scholarships, which favors the appearance of burnout syndrome in these athletes and hinders their continuity in sports, opting to completely devote themselves to the world of work once they have finished their studies (Judge et al., 2012; Åkesdotter et al., 2020). Although these results should be taken with caution, if the objective is for all athletes who pursue the dual career to obtain an academic (university) degree to ensure a future alternative after retiring from sports, a greater provision of scholarships to athletes of different ages and sport modalities should be evaluated.

Considering the limitations of the present study, it is worth noting that this is the first study that addresses barriers, the importance given to academic qualifications and the perception of university dual careers of elite athletes from a multifactorial perspective after the COVID-19 pandemic. It would be interesting for future studies to follow the same line, providing more scientific evidence to determine the changes produced by the COVID-19 pandemic on the perception of university dual career athletes in the long term. As for the main limitations of the study, the subjects analyzed were very heterogeneous in their sociodemographic characteristics (age, sex, and race) and belonged to different sports modalities and universities, which entails completely different adaptations for student-athletes, so these aspects should be considered in future research. Another limitation is that due to the heterogeneity of the sample and the lack of information on the adaptations carried out by the different universities of origin for the success of the dual career, it was not possible to analyze the dimensions referring to tools and tutorship. It is important for future research to collect information on these aspects in different universities and to analyze the student-athletes’ perception of these dimensions. Despite the limitations, the study used a representative sample of collegiate Olympians who participated in the Olympic Games, and is the first scientific investigation that addressed the motivations, barriers and perception of the dual career of these athletes in the aftermath of the COVID-19 pandemic. Therefore, this can be taken as a starting point for future research that intends to address this issue in Olympic athletes who are presently performing their sporting activity.

Regarding the practical implications, the programs developed for university dual career athletes should pay close attention to individual athletes who do not receive any type of scholarships, since they perceive greater barriers. As individual athletes find the most difficulties in reconciling study and training times, a consideration should be given to providing more facilities to these athletes, in terms of time flexibility or changes in the exam schedule, especially when the competition schedule requires continuous travel and long concentrations. In addition, since one of the main reasons that hinder the dual career was the high cost of studies, the possibility that other organizations related to the sports field may grant aid to elite athletes that, at least, cover the cost of university tuition, should be considered. The reduced importance given to grades by student-athletes leads us to consider that academic training is presented as a complement to the sports career, being of vital importance that professionals working with these athletes understand this situation and support them regardless of the time they need to finish their studies. Furthermore, given the large number of athletes who are at the end of their sports career and consider themselves professionals, support programs would need to be implemented to ensure that they do not completely leave the field of sports, but continue collaborating in different sports organizations after retiring from professional sports, as well as the creation of programs aimed at helping these athletes find employment related to their studies at the end of their sporting careers.

## Conclusion

The results of the present study allow us to conclude that pre-Olympic athletes of individual modalities show more barriers than those of team modalities. In addition, athletes who do not receive any scholarships show more barriers and attach less importance to academic qualifications than scholarship athletes. Regarding the reasons for starting the dual career, women study to a greater extent than men because they enjoy it and believe that their studies do not interfere with their sports performance; athletes of individual modalities study to increase their possibilities of working in the future; athletes who do not work perceive more interference between performance and studying; at the beginning of the sports career and at its best stage, athletes perceive a greater interference of their studies with performance, and have a greater intention to continue in the field of sports after their retirement from competitions; professional athletes report that the reconciliation of academic and sports life is difficult, and have more intentions to look for a job than to continue with their sports career after finishing their studies; and athletes who did not receive any type of scholarship perceive a greater interference between their studies and performance, and a greater difficulty in reconciling sports and academic life, but have more expectations of continuing with their sports career after finishing their studies.

## Data Availability Statement

The raw data supporting the conclusions of this article will be made available by the authors, without undue reservation.

## Ethics Statement

The studies involving human participants were reviewed and approved by the Institutional Ethics Committee of the San Antonio de Murcia Catholic University (code: 19/06/2015). The patients/participants provided their written informed consent to participate in this study.

## Author Contributions

AM-O participated in conceptualization, data curation, formal analysis, investigation, methodology, validation, and writing the original draft of the manuscript. AL-A participated in conceptualization, funding acquisition, investigation, project administration, supervision, and writing, review, and editing the manuscript. RV-C participated in conceptualization, data curation, formal analysis, methodology, project administration, validation, and writing the original draft of the manuscript. LA-C participated in conceptualization, data curation, formal analysis, methodology, project administration, visualization, and writing the original draft of the manuscript. JG-R participated in conceptualization, funding acquisition, investigation, project administration, supervision, and writing, review, and editing the manuscript. LM participated in conceptualization, data curation, formal analysis, methodology, project administration, supervision, validation, and writing the original draft of the manuscript. EI participated in conceptualization, project administration, supervision, and writing, review, and editing the manuscript. AS-P participated in conceptualization, funding acquisition, investigation, project administration, supervision, and writing, review, and editing the manuscript. All authors contributed to the article and approved the submitted version.

## Conflict of Interest

The authors declare that the research was conducted in the absence of any commercial or financial relationships that could be construed as a potential conflict of interest.

## Publisher’s Note

All claims expressed in this article are solely those of the authors and do not necessarily represent those of their affiliated organizations, or those of the publisher, the editors and the reviewers. Any product that may be evaluated in this article, or claim that may be made by its manufacturer, is not guaranteed or endorsed by the publisher.
